# *Dictyostelium* spastin is involved in nuclear envelope dynamics during semi-closed mitosis

**DOI:** 10.1080/19491034.2022.2047289

**Published:** 2022-03-17

**Authors:** Ulrike Schweigel, Petros Batsios, Annette Müller-Taubenberger, Ralph Gräf, Marianne Grafe

**Affiliations:** a Department of Cell Biology, University of Potsdam, Institute for Biochemistry and Biology, Potsdam-Golm, Germany; b Department of Cell Biology, LMU Munich, Biomedical Center, Planegg-Martinsried, Germany

**Keywords:** Spastin, LEM-domain, ESCRT, sun1, *dictyostelium*, nuclear envelope, mitosis

## Abstract

Dictyostelium amoebae perform a semi-closed mitosis, in which the nuclear envelope is fenestrated at the insertion sites of the mitotic centrosomes and around the central spindle during karyokinesis. During late telophase the centrosome relocates to the cytoplasmic side of the nucleus, the central spindle disassembles and the nuclear fenestrae become closed. Our data indicate that Dictyostelium spastin (DdSpastin) is a microtubule-binding and severing type I membrane protein that plays a role in this process. Its mitotic localization is in agreement with a requirement for the removal of microtubules that would hinder closure of the fenestrae. Furthermore, DdSpastin interacts with the HeH/ LEM-family protein Src1 in BioID analyses as well as the inner nuclear membrane protein Sun1, and shows subcellular co-localizations with Src1, Sun1, the ESCRT component CHMP7 and the IST1-like protein filactin, suggesting that the principal pathway of mitotic nuclear envelope remodeling is conserved between animals and Dictyostelium amoebae.

## Introduction

In eukaryotes, there are various extents of nuclear envelope (NE) remodeling during mitosis[1]. Animal cells undergo an open mitosis with complete NE breakdown to allow free access for proteins involved in spindle formation and chromosome segregation. After that, NE re-formation occurs during telophase. In contrast, during closed mitosis, as typical for many unicellular eukaryotes, full integrity of the NE including controlled nuclear transport through nuclear pore complexes (NPCs) is preserved throughout mitosis [2]. Between these two extremes there are various forms of semi-closed mitosis with partial disassembly of the NE. Based on ultrastructural and light-microscopic data, in the amoebozoan *Dictyostelium discoideum* the NE remains fully intact, except for two fenestrae harboring the two mitotic centrosomes [3,4]. How the centrosome induces NE fenestration is largely unknown although there is experimental evidence for a role of the centrosomal protein CP75 in this process [5,6]. The process is reminiscent of spindle pole body insertion in fission yeast [7]. Current research suggests that the fenestrae at the centrosome insertion sites, together with partially disassembled NPCs, enable spindle assembly factors, and tubulin dimers to access the nuclear interior [3,8]. Further fenestration occurs only in telophase upon karyokinesis, when abscission of the NEs of the two daughter nuclei takes place around the still existing central spindle [4]. To restore NE integrity, re-sealing of the NE at all sites of fenestration is required for the completion of cell division.

In late mitosis of animal cells, closure of gaps in the re-forming NE requires not only membrane remodeling proteins but also the local disassembly of microtubules, that is, kinetochore microtubules and pole-to-pole microtubules both of which still penetrate these gaps [9]. Membrane remodeling at the NE occurs via the endosomal sorting complex required for transport (ESCRT), and microtubule disassembly is driven by the AAA-ATPase SPASTIN. Recruitment of the latter requires at least the LEM-domain protein LEM2, the ESCRT-III-like proteins CHMP7, and IST1 [10–13]. In mammalian cells the *SPAST* gene yields two major SPASTIN isoforms, called M1, which is anchored to membranes, and the shorter, soluble M87 [14,15]. Mutations in *SPAST* are responsible for spastic paraplegia 4, the most common form of hereditary spastic paraplegia with axonal degeneration and spasticity in the lower extremities [15].

In this work, we present evidence that the processes of NE remodeling during late mitosis of animal cells are already present in amoebozoa. We have focused on a spastin-like protein in *Dictyostelium* (DdSpastin) and its interplay with the LEM/HeH-family protein Src1, an integral inner nuclear membrane protein [16], and with a CHMP7 and IST1 homologue. In addition, we show a novel interaction of spastin with the LINC complex protein Sun1, another inner nuclear membrane protein localizing to the pericentrosomal region [17].

## Materials and methods

### Vectors, strains, and antibodies

Are described in detail in the Supplementary Material and Methods.
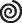


### Protein interaction assays

The BioID assay [18] was performed with isolated nuclei [19] of the DdSpastin-BirA* strain. GFP-Trap® Agarose (Chromotek, Martinsried, Germany) was used for native protein purification and immunoprecipitation of DdSpastin-GFP according to Kastner et al [20]. Proteins in the immunoprecipitation eluate were separated by SDS-PAGE and stained with Coomassie. The band of interest was cut out and analyzed by mass spectrometry after overnight trypsination as published previously [20].

### Microtubule binding and severing assay

Porcine brain tubulin (kind gift from Dr. Günther Woehlke, TUM München, isolated according to Adio et al. [21]) was polymerized in BRB80 buffer [22] supplemented with 1 mM GTP and 10% glycerol for 30 min at 37°C. The microtubules were stabilized by adding a final concentration of 20 µM Taxol (Phytolab, Vestenbersgreuth, Germany). Stabilized microtubules and purified DdSpastin-GFP were diluted in BRB80 supplemented with 20 µM Taxol. Binding and severing was accomplished by incubation of 1 µM each with and without 1 mM ATP for 15 min at room temperature. The reaction mixture was fixed on poly-L-lysine coated coverslips with 4% formaldehyde in 80 mM Pipes pH 6.8 for 5 min and stained with anti-α-tubulin (YL1/2) and anti-rat-Alexa Fluor 594.

### Microscopy

For immunofluorescence probes microscopy the cells were fixed and labeled as described previously [17]. Light microscopy, image processing, and deconvolution with measured point spread functions were performed on a Zeiss CellObserver HS system or an AxioObserver System (Carl Zeiss, Jena, Germany) with Zeiss Axiovision or ZEN-blue software as described recently [23]. For membrane orientation experiments, nuclei of DdSpastin-GFP were isolated and fixed either in the absence or presence of 0.5% Triton X-100 [16].

## Results

**Figure 1.**
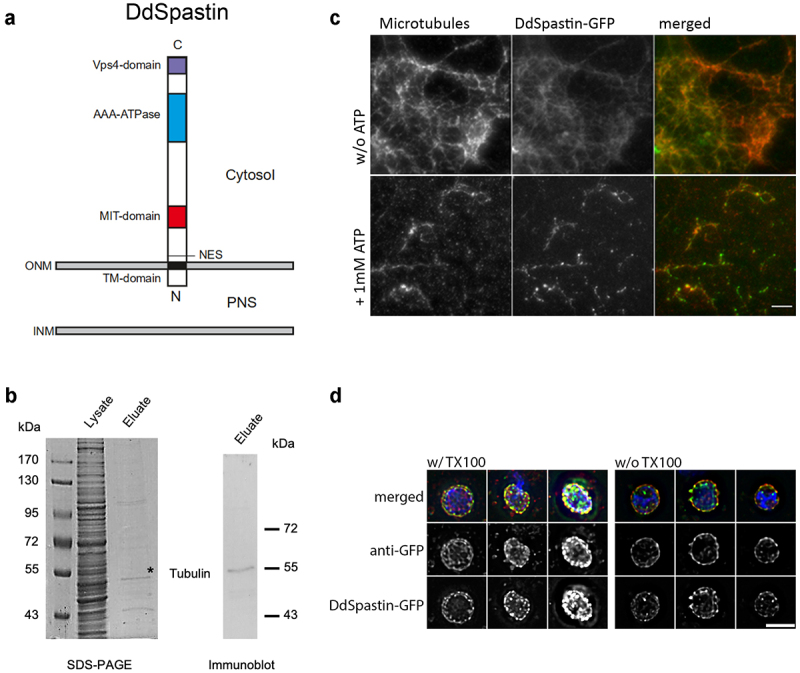
Domain conservation and membrane orientation of DdSpastin. (a) Schematic of DdSpastin domains and membrane orientation by motif predictions using ELM 25, 26. See text for further descriptions; PNS, perinuclear space. (b) Immunoprecipitation using GFP-Trap Agarose beads showing tubulin-binding of DdSpastin-GFP. Proteins in the supernatant (lysate; corresponding to ~10^6^ cells) and the GFP-Trap eluate (corresponding to 1 × 10^7^ cells) were separated by SDS-PAGE, and stained with Coomassie or evaluated by immunoblot staining with anti-β-tubulin; *, this particular band was analyzed by mass spectrometry resulting in a hit for α-tubulin (see table S1). (c) *In vitro* microtubule severing assay. Polymerized porcine brain tubulin and DdSpastin-GFP (green) were incubated with and without 1 mM ATP. The reaction mixture was fixed with formaldehyde on poly-L-lysine coated coverslips and stained with anti-α-tubulin (red). Green spots most likely represent DdSpastin-GFP clusters that have formed via hydrophobic interactions of the transmembrane domains. Bar, 5 µm. (d) Verification of membrane orientation using isolated nuclei from DdSpastin-GFP overexpression cells. Nuclei were fixed with and without Triton X-100 permeabilization. Merged images of three examples each and corresponding single channel images are shown. Bar, 2 μ

We have identified DdSpastin in a BLASTP search by its similarity to human SPASTIN (isoform M1). The *Dictyostelium* gene *DDB_G0287165* encodes a 655 amino acid protein with a calculated molecular mass of 74 kDa and five domains, a transmembrane domain (aa 59–79), a nuclear export sequence (NES; aa 89–96), an MIT (microtubule interacting and transport) domain (aa 169–230) required for recruitment of ESCRT-III proteins, an AAA-ATPase domain (aa 416–552), and a Vps4 oligomerization domain (aa 621–653) (Figure 1(a)). The short N-terminal part upstream of the transmembrane region is predicted to be non-cytosolic (Phobius; [24]). The C-terminal part exhibits a surprising homology to human SPASTIN with an amino acid identity of 57% within the AAA-ATPase and Vps4 domains (aa 348–653). The relationship with spastin homologs of other species is visualized in the phylogenetic tree showing that the amoebozoan spastin is most closely related to opisthokont spastins, as expected (Figure S1). In contrast to the C-terminal half, the N-terminal half including the MIT and transmembrane domains is only weakly conserved, and a microtubule-binding domain (MTBD) of human spastin right upstream from the AAA-ATPase domain [15] is not recognized by sequence pattern predictions. To confirm that DdSpastin is indeed a microtubule-binding protein, we expressed C-terminal GFP-tagged DdSpastin (DdSpastin-GFP) in *Dictyostelium* and affinity purified the fusion protein using GFP-trap beads. The eluate was tested for co-purification of tubulin by mass spectrometry and Western blotting with a monoclonal anti-β-tubulin antibody. Mass spectrometry revealed that the eluate contained α-tubulin (Table S1) and the Western blot revealed that b-tubulin clearly co-purified with DdSpastin-GFP (Figure 1(b)). In a control experiment with a strain expressing unfused GFP, protein staining of GFP-trap eluates revealed no unspecific bands in the range from 20 kDa and higher (Figure S2). Moreover, the purified DdSpastin-GFP was used in a functional *in vitro* assay with porcine brain microtubules. In this assay, DdSpastin co-localized with microtubules and, after addition of ATP, was capable of microtubule severing (Figure 1(c)). The affinity of DdSpastin to microtubules *in vivo* was investigated in a further *Dictyostelium* strain expressing green-fluorescent DdSpastin without the transmembrane domain (DdSpastin∆TM-GFP) together with mScarlet-α-tubulin (mScarlet-TubA) to yield red-fluorescent microtubules (Figure S3). In addition to cytosolic foci, DdSpastin∆TM-GFP clearly localized along the microtubules, proving the microtubule-binding capabilities of DdSpastin. DdSpastin∆TM-GFP expressing cells were about two times smaller than control cells and more than twice more susceptible to damages during fixation (Figure S3).

**Figure 2.**
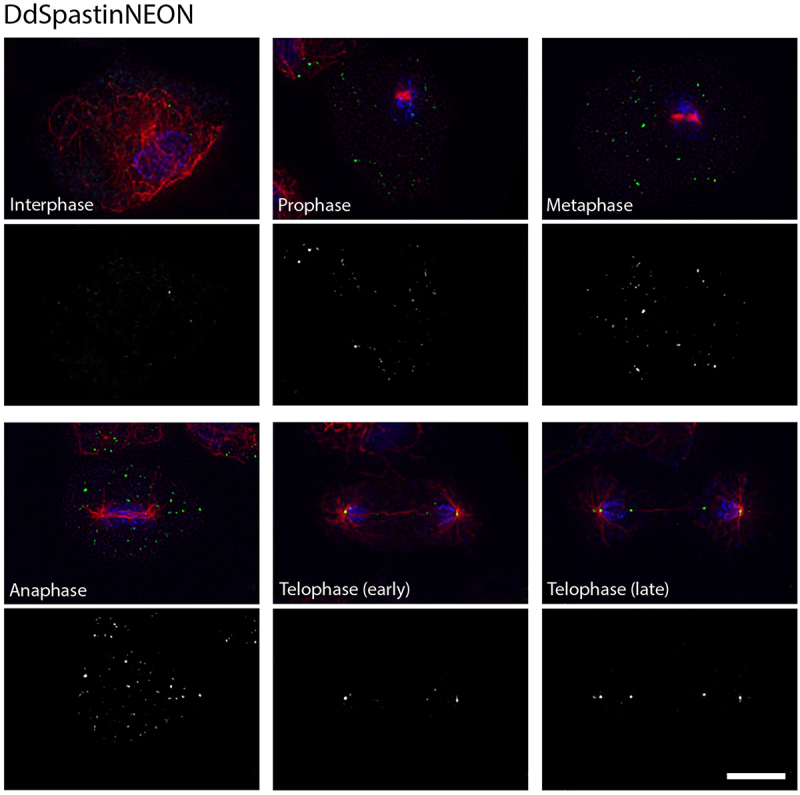
Localization of DdSpastin-NEON (knock-in). Cells were fixed with glutaraldehyde and stained with DAPI(blue), and anti-α-tubulin (red). DdSpastin-NEON (green) accumulated at spindle Poles beginning in early telophase and in late telophase at the central spindle. The DdSpastin-NEON channel alone is shown below the merged images. A quantitative evaluation of all investigated cells is given in Table S2. Bar, 5 μm.

Expression of DdSpastin-GFP also allowed us to examine the membrane orientation of the fusion protein. Nuclei isolated from DdSpastin-GFP overexpression cells were fixed with and without Triton X-100 permeabilization and labeled with anti-GFP and secondary Alexa Fluor 568 conjugated antibodies (red). DdSpastin-GFP was enriched at the NE. Anti-GFP antibodies stained NEs equally well with and without Triton X-100 permeabilization, confirming the expected orientation with the C-terminus facing the cytosol (Figure 1(d)). Since in the DdSpastin-GFP and DdSpastin∆TM strains the fusion protein is expressed in addition to endogenous DdSpastin, we created a DdSpastin-NEON knock-in strain to avoid misinterpretation by protein overexpression artifacts. Here, the DdSpastin coding sequence is replaced by a sequence encoding the DdSpastin-NEON fusion protein under the control of the endogenous promoter, which should warrant expression of wild-type protein levels. Green-fluorescent NEON was chosen for its superior brightness, and C-terminal fusion was preferred since previous experiments with N-terminal tagging had revealed unwanted cleavage downstream of the tag (see description of BirA* constructs below). In interphase cells DdSpastin-NEON was present in a few spots some of which localized at the NE but also elsewhere (Figure 2). However, during mitosis, beginning in early telophase, DdSpastin-NEON was clearly localized at the spindle poles, and later also at the central spindle close to the equator. Especially in late telophase DdSpastin-NEON localization at the central spindle was associated with the new daughter nuclei, in discernible spots vis-a-vis from the spindle poles (Figure S4, Movie S2; A quantitative evaluation of all investigated cells is given in Table S2). The results provided the first indication that DdSpastin may be involved in the closure of the NE fenestrae by scission of the central spindle at these sites.

**Figure 3.**
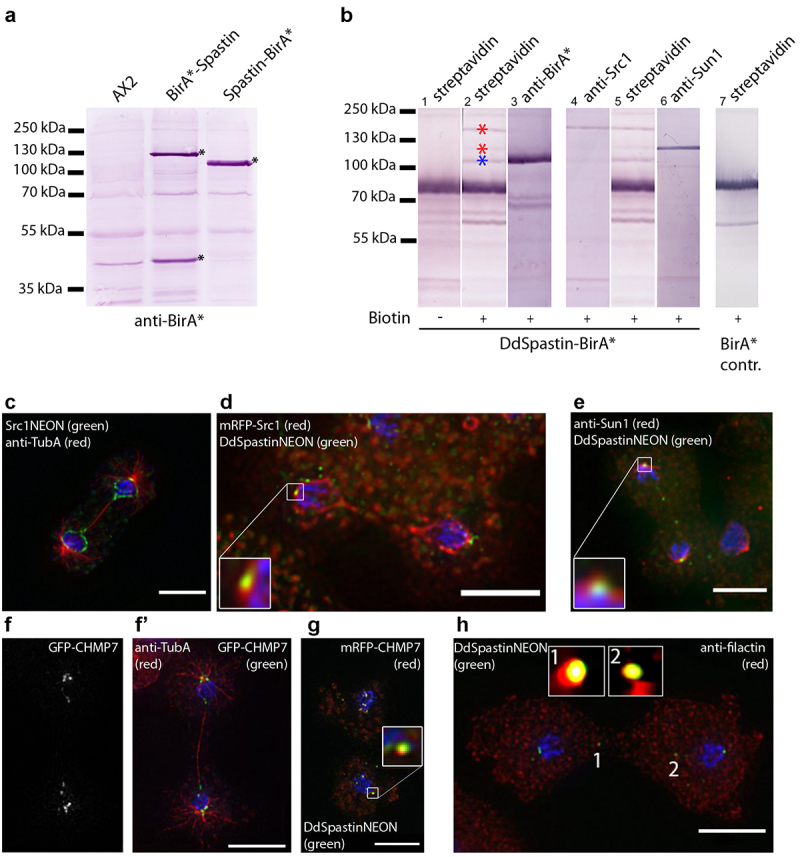
DdSpastin interactions and co-localizations. (a) Immunoblot of whole cell extracts of AX2 control cells, BirA*-DdSpastin cells and DdSpastin-BirA* cells stained with anti-BirA* antibodies. Fusion protein bands and BirA* with signal peptide are labeled with an asterisk. (b) BioID with nuclear extracts of DdSpastin-BirA* (lane 1–6) and negative control BirA* cells (lane 7). Western blots were stained with alkaline phosphate conjugated to the antibodies/protein stated on top. The interactors Src1 and Sun1 are labeled with red asterisks, DdSpastin-BirA* is labeled with a blue asterisk (lane 2). Lane 1 control w/o biotin incubation and lane 7 BirA* control show no specific bands at these positions. (c-h) Fluorescence microscopy of the strains stated in the figures. Cells were fixed with either glutaraldehyde (c-g) or methanol (h), and additionally labeled with DAPI (blue). Close-ups show co-localization (d, e, g, h). GFP-CHMP7 labeling is shown in a single channel because this protein has not been previously published (f). Bar, 5 μm.

Since proximity-dependent biotin identification (BioID) has been the most reliable method to detect protein-protein-interactions in *Dictyostelium* [18], we created both N- and C-terminally tagged versions of DdSpastin with the BirA*-biotinylase. Due to the R118G point mutation this enzyme biotinylates neighboring lysine residues in a promiscuous manner. As successful biotinylation requires a close distance of <10 nm [27], that is, comparable to a FRET assay, this method preferably detects direct-binding partners. Successful expression of fusion proteins was examined by Western blotting (Figure 3(a)). While in C-terminally tagged DdSpastin-BirA*, anti-BirA* antibodies detected one main band of the expected size, two bands of ~120 kDa and ~45 kDa, respectively, were labeled in N-terminally tagged BirA*-DdSpastin. A very similar result was observed with GFP replacing BirA* as the fusion tag (not shown). We conclude that DdSpastin is a type I membrane protein with a N-terminal signal sequence that is cleaved off during translation at the endoplasmic reticulum. Upon N-terminal tagging, the cleavage site could be too far downstream to be effectively interpreted by the signal peptidase, giving rise to the two bands labeled by anti-BirA, that is, the complete fusion protein and BirA* with the signal sequence at its C-terminus. This interpretation is corroborated by the observation that the DdSpastin-BirA* exhibits a slightly smaller apparent molecular mass, most likely due to truncation after cleavage of the N-terminal signal sequence. Having in mind the great variability of N-terminal signal sequences we consider this to be the most likely scenario, although the signal peptide prediction programs employed by Uniprot (https://www.uniprot.org) failed to detect one in DdSpastin. For that reason, we exclusively used the C-terminal fusion strains. Isoforms due to different internal translation initiation sites are unlikely, since then the multiple-band pattern would be expected also with the C-terminal tag.

Employing proximity-dependent BioID, we isolated DdSpastin-BirA* by streptavidin-affinity chromatography and analyzed it by Western blotting with antibodies directed against prospective-binding partners [16] alongside with streptavidin-alkaline phosphatase(Figure 3(b)). Since in animal cells, a LEM-domain protein is involved in NE dynamics during spastin-mediated microtubule severing [10–13], we investigated the relationship between Src1, the only *Dictyostelium* orthologue of the HeH/LEM-domain family [16] and DdSpastin. On split lanes both conjugates clearly stained the same band corresponding to Src1, demonstrating that DdSpastin-BirA* had biotinylated Src1 (lane 4 and 5). In addition, BioID blots always show self-biotinylation of the tagged protein, which can be used as a positive control (lane 2 blue asterisk, and lane 3 stained with anti-BirA*). The negative control from a strain expressing only BirA* solely shows bands corresponding to endogenously biotinylated mitochondrial proteins (lane 7, [18]). The sample without added biotin shows the inducibility of the process (lane 1). A Src1-NEON fusion protein expressed in a corresponding knock-in strain localized to the NE and, after rupture of the NE bridge between the two separating daughter nuclei, was also found at the site of the NE fenestrae around the central spindle and centrosomes (Figure 3(c)). This indicates a co-operation of Src1 and DdSpastin in the closure of the NE fenestrae. The DdSpastin-Src1 interaction is also mirrored by occasional co-localization of both proteins during late mitosis (Figure 3(d) and S5, Movie S3).

Another biotinylated protein band of ~110 kDa identified after streptavidin stainings was the NE protein Sun1 (Figure 3(b), lane 5 and 6). Sun1 is required to maintain the linkage of the centrosome to the NE. During mitosis Sun1 is concentrated at the centrosomal fenestrae and forms a collar around the NE-embedded centrosome [17]. Co-localization of Sun1 with DdSpastin-NEON at these sites during late mitosis agrees with the BioID interaction profile observed in this study (Figure 3(e)). Due to the relevance of the ESCRT-III-like proteins CHMP7 and IST1 for SPASTIN recruitment in addition to LEM2 [12], we looked for co-localization of the corresponding *Dictyostelium* orthologues with DdSpastin. We expressed the *Dictyostelium* CHMP7 orthologue (*DDB0266400*, approx. 56 kDa) with an N-terminal mRFP tag in the DdSpastin-NEON knock-in strain. Fluorescence microscopy revealed co-localization of CHMP7 and DdSpastin at the spindle poles, and at spots in the region of the central spindle (Figure 3(g) and S6, Movie S4). CHMP7 localization at the spindle and the poles was confirmed in microtubule stainings in cells expressing GFP-CHMP7 (Figure 3(f)). Furthermore, we stained the IST1-domain containing protein filactin with a specific monoclonal antibody [28,29]. Filactin, however, was absent from the poles but co-localized with DdSpastin-NEON in discrete spots at the spindle (Figure 3(h)).

## Discussion

Here, we provide the first characterization of spastin in *Dictyostelium* amoebae. Regarding its domain organization DdSpastin is most reminiscent of the M1 isoform of human spastin. M1 is characterized by a hydrophobic domain at its N-terminus that is predicted to form a hairpin loop membrane domain [30]. In *Dictyostelium,* this part appears to be replaced by an α-helical transmembrane domain. Our protein expression studies indicate that the mature DdSpastin protein is oriented with its C-terminal end toward the cytoplasm, and that an N-terminal, soluble part upstream from the transmembrane domain is cleaved off during protein processing at the endoplasmic reticulum. While the C-terminal half of the protein including the AAA-ATPase domain is highly conserved, the microtubule-binding domain is not detected by regular protein sequence annotation tools. Despite of that, DdSpastin clearly acts as a microtubule-associated ATPase. We show that the purified protein binds and severs porcine brain microtubules *in vitro* and that DdSpastin-NEON localizes to the central mitotic spindle and to spindle poles. Moreover, DdSpastin∆TM-GFP resembling the M87 spastin isoform without a transmembrane domain localized clearly along microtubules. The observation that DdSpastin∆TM-GFP expressing cells appeared smaller and were more susceptible to mechanical damage during fixation could be interpreted as a result of shortened or destabilized microtubules and indicates that the fusion protein is functional.

**Figure 4.**
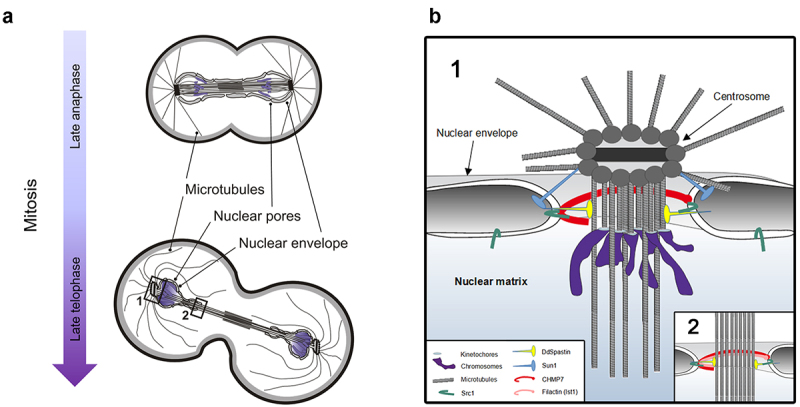
Schematic illustration of *Dictyostelium* cell in late mitosis (a) and corresponding nuclear envelope fenestrae in late telophase. Centrosomes are still inserted in the NE in late anaphase and leave the NE in late telophase. (b) Enlargement with putative proteins involved in closure of the membrane fenestrae at the spindle Poles (1) and central spindle (2) in *Dictyostelium*. Inspired by Sundquist& Ullman (2015) [9]. See text for further details.

While DdSpastin-NEON expressed at endogenous levels showed no specific localization from interphase to mid mitosis, we observed distinct localizations during late mitosis. Starting with late anaphase DdSpastin-NEON accumulated at spindle poles, and in late telophase also at the central spindle. At this point, the mitotic centrosomes leave their fenestrae [31], and the NE fenestrae of both daughter nuclei at the poles and around the central spindle are required to be closed (Figure 4(a)). However, a prerequisite for this process is the severing of the penetrating microtubules. DdSpastin is the perfect candidate protein to fulfill this function. The principal processes of NE and spindle dynamics during late mitosis should be the same in open and semi-closed mitosis, since also in open mitosis the NE has largely re-formed at this point, with the exception of fenestrae at the poles and the spindle [9]. According to the current hypothesis, SPASTIN severs the microtubules penetrating the NE fenestrae, while recruitment of ESCRT-III subunits to the membrane at the fenestrae results in a narrowing helical ESCRT-III assembly and remodeling of these ESCRT-III filaments by the AAA-ATPase Vps4, which finally drives closure of the fenestrae [32,33]. The interaction of CHMP7 and LEM-domain proteins leads to recruitment of the ESCRT-III complex in open and closed mitosis [12], and also in organisms with semi-open mitosis, both proteins are required for correct nuclear membrane re-sealing [34]. The presence of putative orthologues of LEM2, SPASTIN, CHMP7, and IST1 at the fenestrae in *Dictyostelium* and the presence of further orthologues of the ESCRT-III family and Vps4 [35] in the *Dictyostelium* genome strongly suggests that this process is widely conserved throughout eukaryotic phylae. Yet there may be slight differences in the specific requirements for fenestra closing at the poles and the spindle. We observed the IST1-like protein filactin at the spindle but failed to detect it at the poles. Filactin is a hybrid protein containing an actin-like domain and an IST1-like domain. It was characterized as a component of intranuclear actin rods that are formed in *Dictyostelium* amoebae in response to chemical or mechanical stress conditions [29]. Otherwise, its function is not well understood. The novel localization at the late mitotic spindle indicates a yet uncharacterized role of filactin in the closure of NE fenestrae at the central spindle. In contrast to filactin, Sun1 is concentrated around the mitotic centrosomes, but absent from the spindle. The intimate interaction between Sun1 and DdSpastin has not been shown before in any other species. It indicates that Sun1 is not only a key player in centrosome insertion but also in centrosome extrusion to the cytosolic side of the NE (Figure 4(b)). Here, Sun1 could be involved in the recruitment of DdSpastin, which then severs the centrosome-kinetochore microtubules as a prerequisite for NE fenestrae closing. At the opposite sides, that is, around the central spindle, NE remodeling could also involve disassembly and re-assembly of the nuclear pore complex, as it was shown in fission yeast [2,36, 37–51]. Differences between the closure of NE fenestrae at the poles and around the spindle could also arise from the vicinity of kinetochores to the closure site at the poles versus that of midbody-associated proteins to closure sites at the central spindle. A full understanding of the dynamic processes at the NE during semi-closed mitosis will certainly require characterization of the other players involved. The finding that the spastin-dependent NE remodeling processes are conserved also in amoebozoae, and the good genetic and microscopical accessibility of *Dictyostelium* amoebae make these cells an attractive model to study the etiology of spastic paraplegia 4.

## Supplementary Material

Supplemental Material

## Data Availability

The authors confirm that the data supporting the findings of this study are available within the article and its supplementary materials.
