# Supplementary material for: Dictyostelium spastin is involved in nuclear envelope dynamics during semi-closed mitosis
Source: Nucleus. 2022 Mar 17;13(1):144–54. doi: 10.1080/19491034.2022.2047289 (PMC8932920; doi:10.1080/19491034.2022.2047289)
Supplement: Supplemental Material [file KNCL_A_2047289_SM1898.docx]

**Supplemental Material**

**Table S1:** Mass spectrometric analysis of the DdSpastin-GFP pulldown (Fig. 1B lane *)

| # | Identified Proteins (20) | Acc. N. | MW | Prot. Id. Pr. | Total Sp. C. |
| --- | --- | --- | --- | --- | --- |
| 1 | elongation factor 1 alpha | gi\|66824969 (+3) | 50 kDa | 100% | 120 |
| 2 | leucine aminopeptidase | gi\|66814716 | 56 kDa | 100% | 36 |
| 3 | peptidase M16 family protein | gi\|66803202 | 48 kDa | 100% | 5 |
| 4 | alpha tubulin | gi\|66806671 | 51 kDa | 100% | 7 |
| 5 | cortexillin II | gi\|66818421 | 50 kDa | 100% | 2 |
| 6 | group IV decarboxylase | gi\|66813288 | 52 kDa | 100% | 3 |
| 7 | RhoGAP domain-containing protein [ | gi\|66809573 | 44 kDa | 100% | 4 |
| 8 | actin bundling protein | gi\|66804885 | 51 kDa | 100% | 3 |
| 9 | NAD-dependent glutamate dehydrogenase | gi\|66806963 | 55 kDa | 100% | 3 |
| 10 | protein serine/threonine kinase | gi\|66822261 (+1) | 49 kDa | 100% | 1 |
| 11 | 26S proteasome ATPase 2 subunit | gi\|66818341 | 47 kDa | 100% | 2 |
| 12 | histone H4 | gi\|66818038 | 12 kDa | 100% | 2 |
| 13 | hypothetical protein DDB_G0280585 | gi\|66813768 | 36 kDa | 99% | 1 |
| 14 | hypothetical protein DDB_G0268060 | gi\|66828341 | 90 kDa | 99% | 1 |
| 15 | eukaryotic translation termination factor 1 | gi\|66805833 (+1) | 49 kDa | 99% | 1 |
| 16 | aldehyde dehydrogenase | gi\|66805929 | 55 kDa | 99% | 1 |
| 17 | repC-binding protein A | gi\|66808013 (+1) | 37 kDa | 99% | 1 |
| 18 | COP9 signalosome complex subunit 1 | gi\|66810608 | 53 kDa | 99% | 1 |
| 19 | tyrosine-tRNA ligase | gi\|66818755 | 44 kDa | 99% | 1 |
| 20 | AAA ATPase domain-containing protein | gi\|66807301 | 74 kDa | 99% | 1 |

Acc. N.: Accession Number

MW : Molecular Weight

Prot. Id. Pr.: Protein Identification Probability

Total Sp. C.: Total Spectrum Count

**Table S2:** Localization of DdSpastin-NEON (knock-in) in fixed cells according to Figure 2.

|  | Mitotic stage | CF [µm] | SP to SP [µm] | Spot at SP | Spot at CS |
| --- | --- | --- | --- | --- | --- |
| 001 | Prophase | 13,19 | 0,68 | no | no |
| 002 | Metaphase | 12,25 | 2,99 | no | no |
| 003 | Anaphase | 8,52 | 4,35 | no | no |
| 004 | Anaphase | 11,57 | 7,06 | yes | no |
| 005 | Telophase | 8,31 | 10,22 | yes | no |
| 006 | Telophase | 6,84 | 10,71 | yes | yes |
| 007 | Telophase | 5,78 | 10,60 | yes | yes |
| 008 | Cytokinesis | 5,37 | 12,88 | yes | yes |
| 009 | Cytokinesis | 4,20 | 16,93 | yes | yes |
| 010 | Cytokinesis | 3,72 | 11,34 | yes | yes |
| 011 | Cytokinesis | 3,06 | 12,74 | yes | yes |
| 012 | Cytokinesis | 2,96 | 13,18 | yes | yes |
| 013 | Cytokinesis | 2,84 | 13,97 | yes | yes |
| 014 | Cytokinesis | 2,25 | 14,12 | yes | yes |
| 015 | Cytokinesis | 1,91 | 10,36 | yes | yes |
| 016 | Cytokinesis | 1,74 | 14,58 | yes | yes |
| 017 | Cytokinesis | 1,56 | 8,00 | yes | yes |
| 018 | Cytokinesis | 1,52 | 10,45 | yes | yes |
| 019 | Cytokinesis | 1,48 | 11,85 | yes | yes |
| 020 | Cytokinesis | 1,40 | 13,70 | yes | yes |
| 021 | Cytokinesis | 1,28 | 12,23 | yes | yes |
|  | | | | | |
| CF: Cleavage furrow  SP: Spindle pole  CS: Central spindle | | | | | |


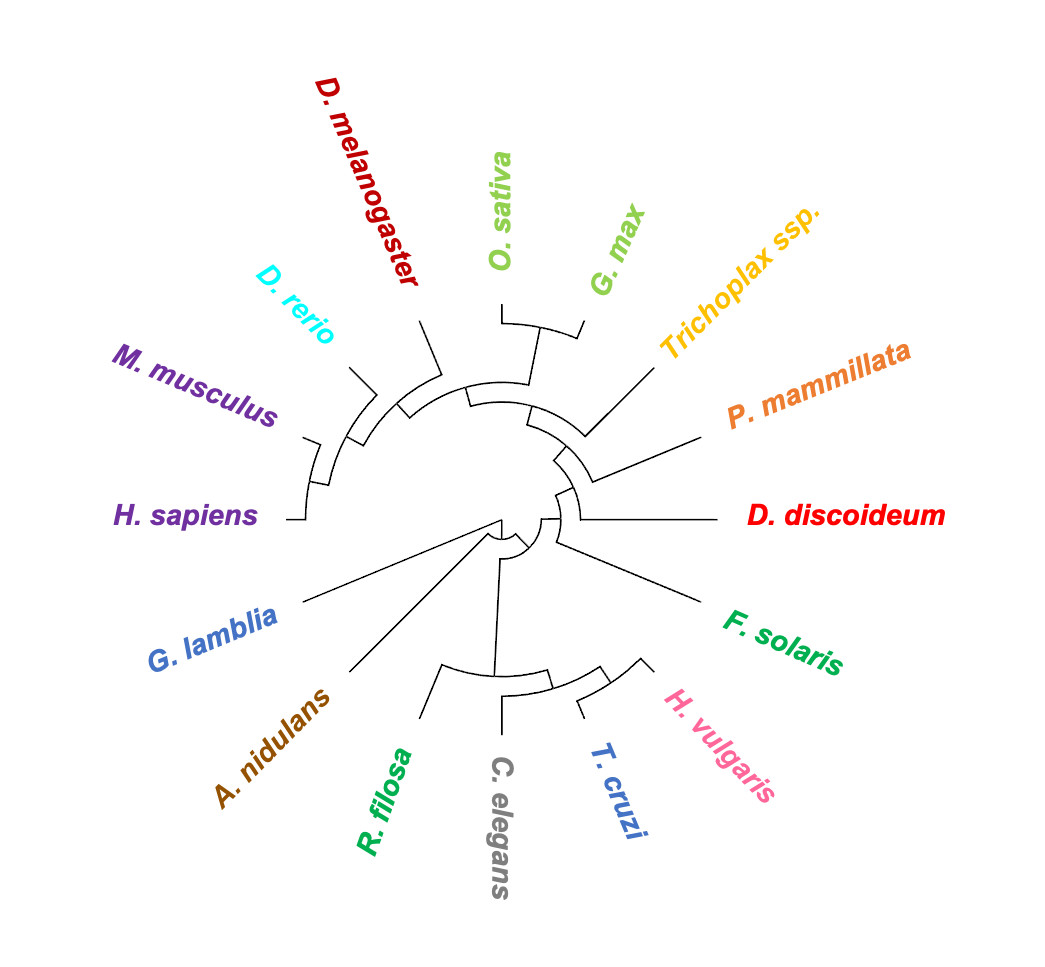
**Figure S1**

**
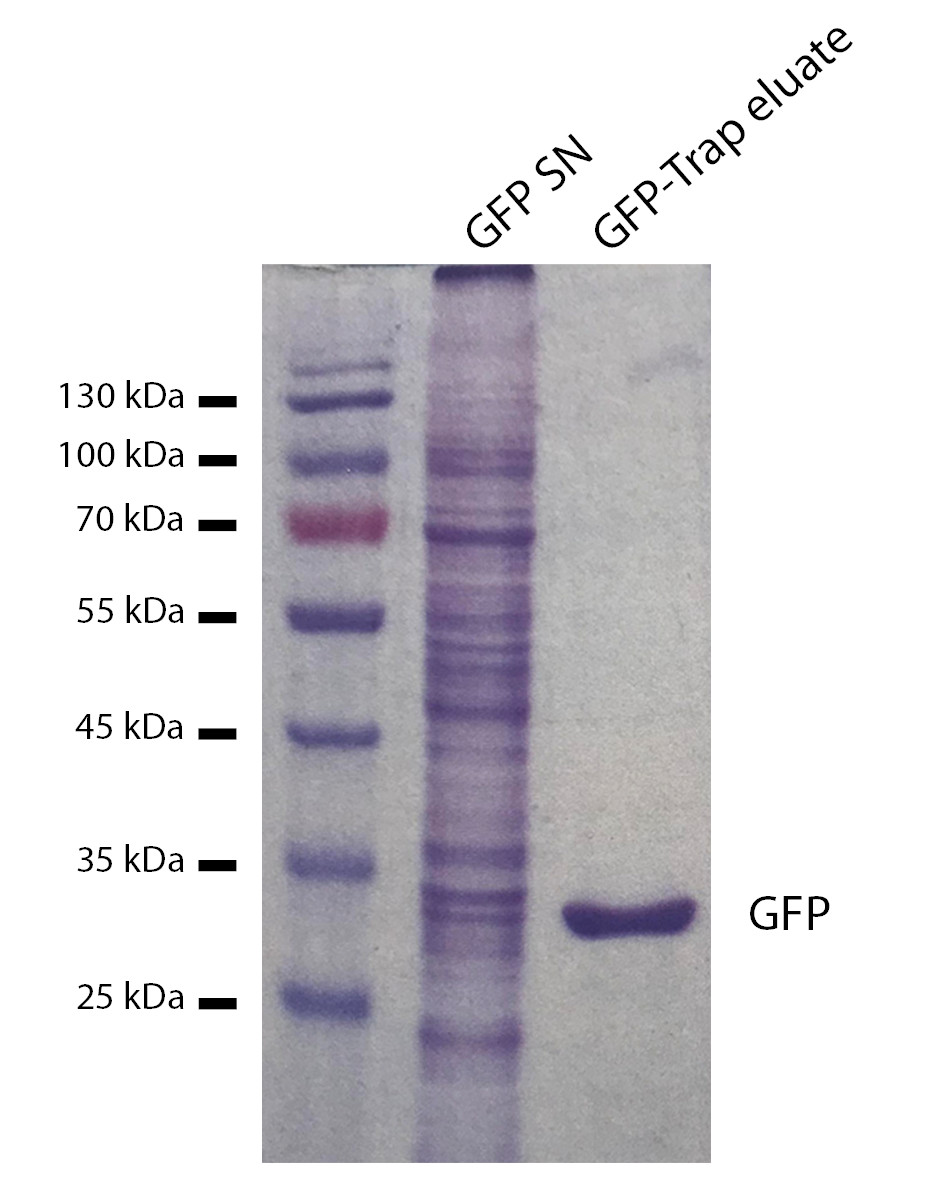
**

**Figure S2**


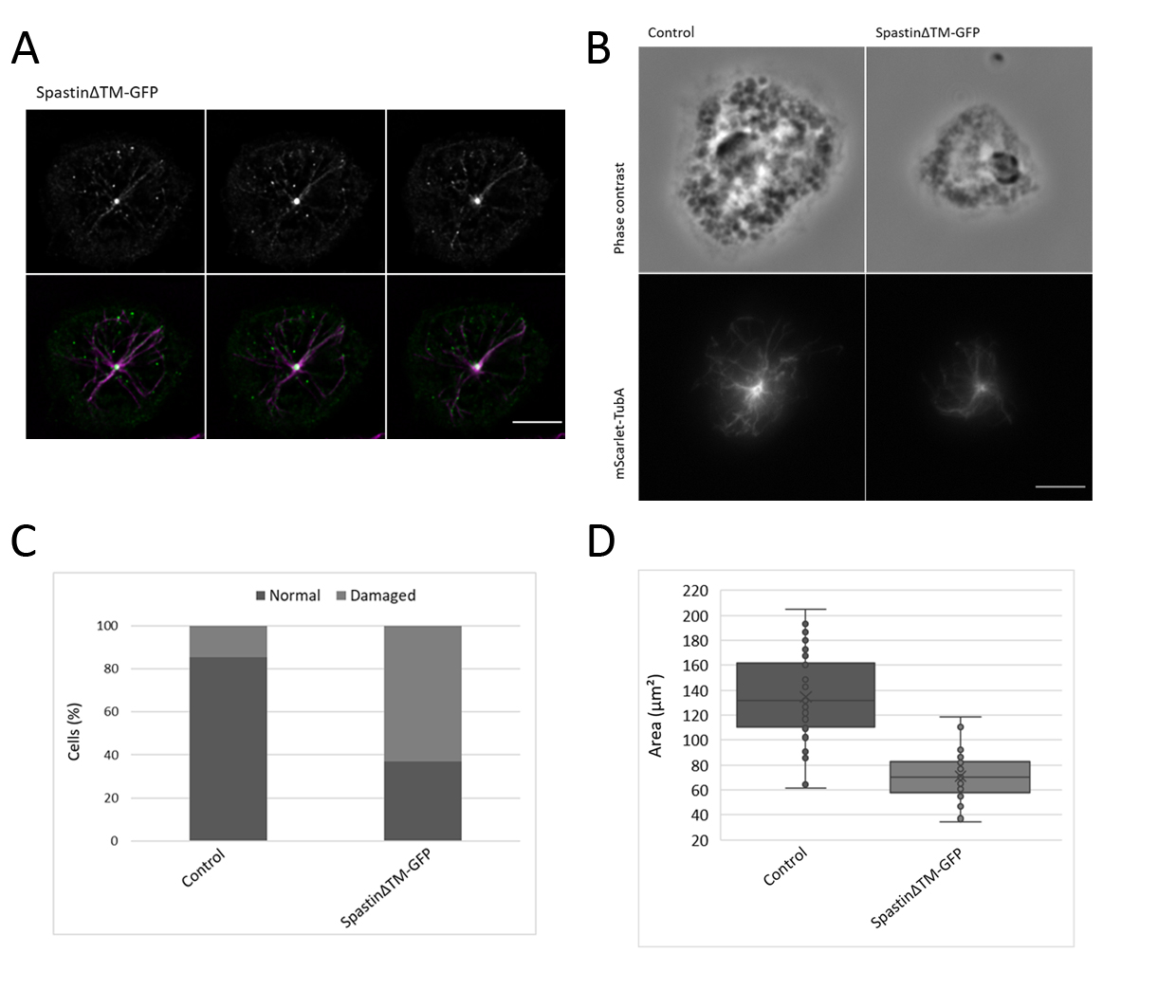


**Figure S3.**

**
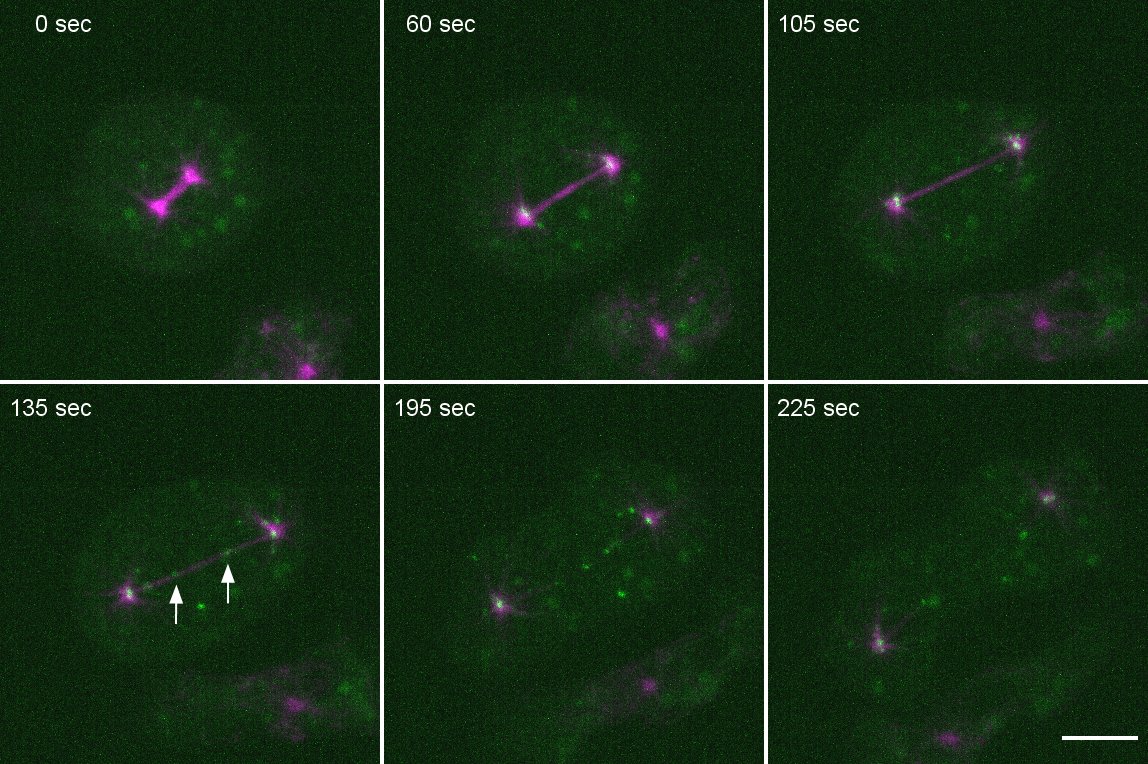
**

**Figure S4/Movie S2:**

**
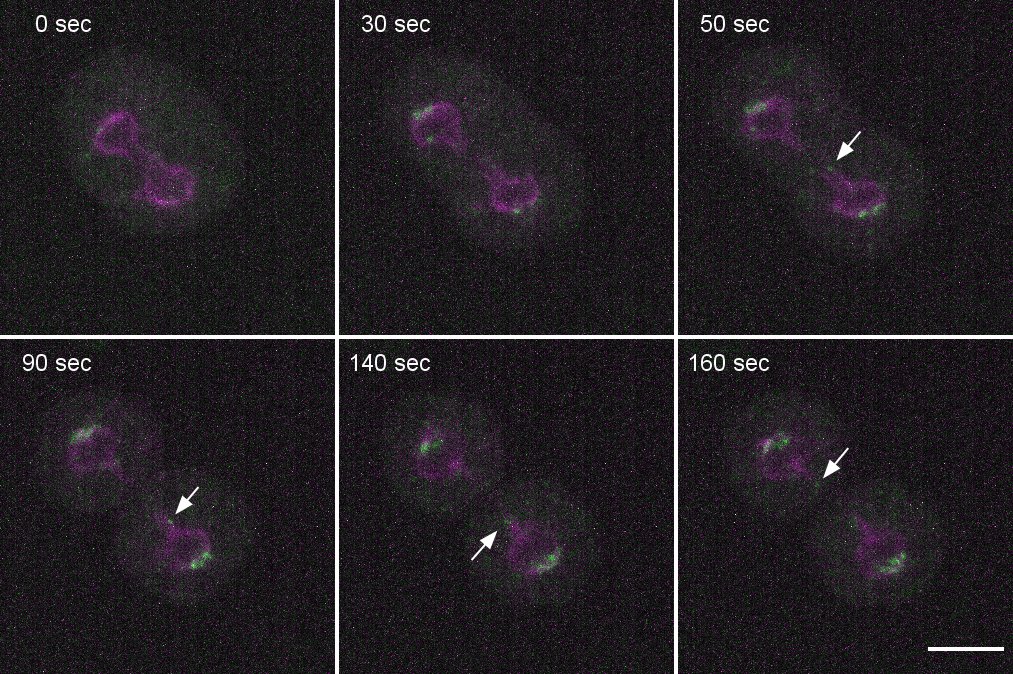
**

**Figure S5/Movie S3:**


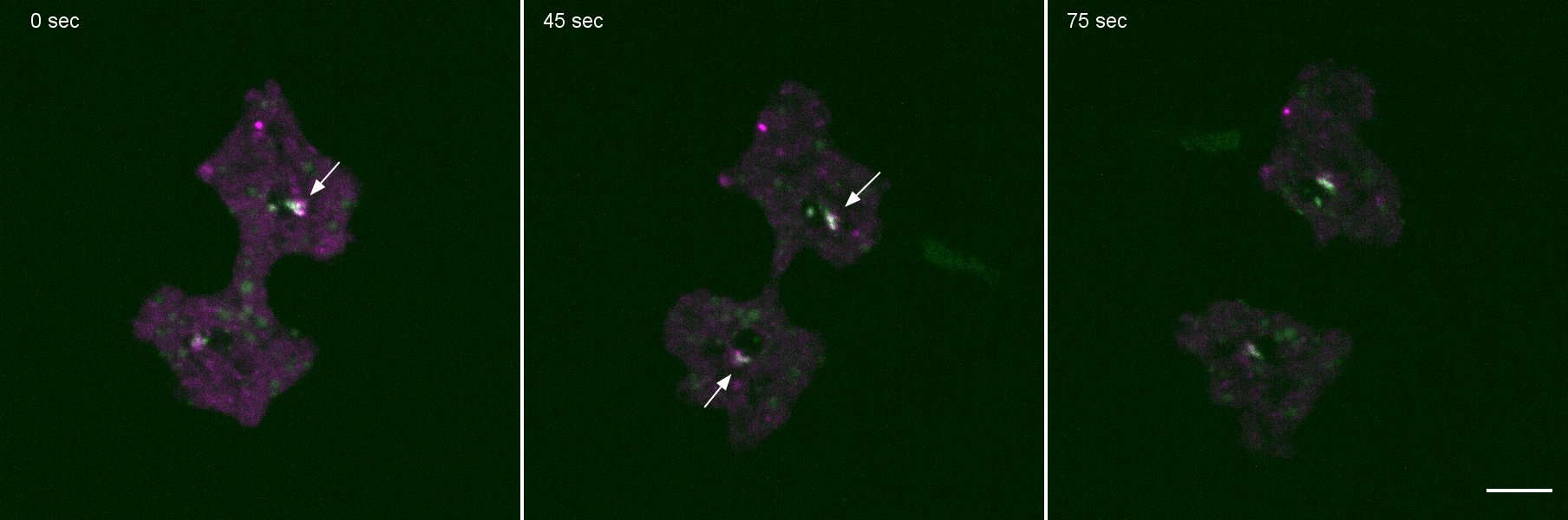


**Figure S6/Movie S4:**

# **Supplementary Figure Legends**

**Figure S1.** Phylogenetic tree of Spastin proteins identified by blast searches at NCBI. Entire sequences were calculated by COBALT with neighbor joining algorithm ^36^. Taxa are color coded: Excavata (blue; T. cruzi ESS62028.1; G.lamblia EFO62094.1), Archaeplastida (light-green; G. max, XP_003552348.1; O.sativa, BAD44799.1), SAR-group (dark-green; F.solaris GAX24550.1; R.filosa, ETO12522.1), Fungi (brown; A. nidulans, XP_663867), Placozoa (yellow; Trichoplax ssp. RDD44344.1), Cnidaria (rose; H. vulgaris, CDG66790.1), Nematoda (grey; C. elegans, NP_741586.1), Arthropoda (dark-red; D. melanogaster, NP_001303437.1), Tunicata (orange; P. mammillate, CAB3266511.1), Craniata (cyan; D. rerio, NP_998080.2), Amoebozoa (red; *D. discoideum, DDB0231319), Mammals (purple; M. musculus, NP001344667; H. sapiens, NP955468).

**Figure S2.** Immunoprecipitation of GFP from Dd wild-type cells using a GFP-Trap kit (Chromotek, Martinsried, Germany). Supernatant (SN) and immunoprecipitated proteins (eluate) were separated by SDS–PAGE and stained with Coomassie blue. Identified protein is indicated on the right (for Western blot analysis see (^20^ Fig.6A).

**Figure S3:** DdSpastinΔTM co-localizes with microtubules and the centrosome. DdSpastinΔTM-GFP expression results in cell damages during fixation and a reduction of cell size. (A) Z-slice of a mScarlet-TubA (magenta) cell expressing DdSpastinΔTM-GFP (green and top row) fixed with glutaraldehyde. See **Movie S1** for the whole image stack. (B) top: phase contrast image (one focal plane); bottom, mScarlet-TubA (maximum intensity projection of 15 focal planes). Cells were fixed with glutaraldehyde. (C) Control: mScarlet-TubA cells (n=48); appearance of cell shape and cytoplasm in phase contrast after fixation. Cells expressing DdSpastinΔTM-GFP are more sensitive to mechanical damage during fixation (n=38). (D) Control: mScarlet-TubA cells (n=32); DdSpastinΔTM-GFP in mScarlet-TubA cells (n=32); cell sizes measured by area calculation of phase contrast images using Fiji ^37^.

**Figure S4/Movie S2.** Co-localization of mScarlet-TubA (magenta) and DdSpastin-NEON (green). Live cell imaging with cells under agar overlay ^38^ was performed with an Olympus Ixplore SpinSR microscope (Olympus Corporation, Shinjuku, Tokyo, Japan) equipped with confocal spinning disk unit CSU-W1 SoRa (Yokogawa Electric, Musashino, Tokyo, Japan) and Orca-Flash 4.0 V3 camera (Hamamatsu Photonics, Hamamatsu, Japan) using a 100x objective (UPLSAPO 100X /1,35). Recording starts in Anaphase (0 sec). DdSpastin-NEON (green) accumulates at spindle poles beginning in early telophase (from 60 sec) and later at the central spindle (arrows, from 135 sec). Bar, 5 µm.

**Figure S5/Movie S3.** Co-localization of mRFP-Src1 (magenta) and DdSpastin-NEON (green). Live cell imaging with cells under agar overlay ^38^ was performed with an Olympus Ixplore SpinSR microscope (Olympus Corporation, Shinjuku, Tokyo, Japan) equipped with confocal spinning disk unit CSU-W1 SoRa (Yokogawa Electric, Musashino, Tokyo, Japan) and Orca-Flash 4.0 V3 camera (Hamamatsu Photonics, Hamamatsu, Japan) using a 100x objective (UPLSAPO 100X /1,35). Recording starts in Anaphase (0 sec). DdSpastin-NEON (green) accumulates at spindle poles beginning in early telophase (from 30 sec) and later at the central spindle (arrows, from 50 sec). Bar, 5 µm.

**Figure S6/Movie S4.** Co-localization of mRFP-CHMP7 (magenta) and DdSpastin-NEON (green). Live cell imaging with cells under agar overlay ^38^ was performed with an Olympus Ixplore SpinSR microscope (Olympus Corporation, Shinjuku, Tokyo, Japan) equipped with confocal spinning disk unit CSU-W1 SoRa (Yokogawa Electric, Musashino, Tokyo, Japan) and Orca-Flash 4.0 V3 camera (Hamamatsu Photonics, Hamamatsu, Japan) using a 100x objective (UPLSAPO 100X /1,35). Recording starts in Cytokinesis (0 sec). Selected time points show co-localization of CHMP7 and DdSpastin at the spindle poles (arrows). Bar, 5 µm.

**Supplementary Material and Methods**

**Strains and Vectors**

All strains are based on AX2 wild type strains. Cells were cultured in HL5c medium (Formedium, Hunsanton, UK) and transformed by electroporation according to ^39^. For the C-terminal DdSpastin-GFP overexpression construct, the full-length sequence of DdSpastin (DDB_G0287165) was either cloned into the *Eco*RI-site of the GFP expression vector pDEX27 ^40^ for membrane orientation experiments. The GFP control strain is described in ^20^. For the DdSpastinΔ1−71-GFP overexpression construct (SpastinΔTM-GFP) the coding sequence of DdSpastin starting from base 217 (aa 72) was cloned into the C-terminal GFP-expression vector p1ABsr8 ^41^ using *Kpn*I/*Bam*HI linker primers. For the DdSpastin-NEON knock-in construct pIS1307, the vector was build according to the C-terminal knock-in construct pIS1121 ^42^ where GFP was replaced with a codon-optimized mNeonGreen ^43^. The inserted DdSpastin sequences were amplified with linker primers T62 (TAGTGGATCCcattaagagaattaaattataaagg)/T63(AATTCCTGCAGgagttgaatatgaatttgatattgg) for the C-terminal coding sequence) and T64 (ccatgaattcAATAGTTCCAAACTTTTGATTCC)/T65 (GCTGGGTACCGAAATTGATTCATTGCTAACTGAACG) for the 3′ untranslated sequence. For the Scarlet-TubA construct, α-tubulin with an N-terminal actin6 promoter/GFP/polylinker cassette ^44^ was cloned into the pLPBLP vector ^45^ and GFP was replaced subsequently with mScarlet ^46^ using the *NheI/SalI* restriction sites. For the BioID constructs, the full-length sequence of DdSpastin was cloned into the C-terminal BirA-R118G vector pIS1000 using the *Kpn*I/*Bam*HI restriction sites ^42^ and the N-terminal BirA-R118G vector pPB87 using the *Bam*HI/*Sal*I restriction sites ^47^. For the BirA* control pPB87 was used. The BirA* antibody was obtained by custom immunization of a rabbit (Preclinics, Potsdam, Germany) using recombinant BirA* expressed via a modified pMALc2 vector (NEB, Frankfurt, Germany) ^48^. The Src1-NEON knock-in construct was build according to the DdSpastin-NEON knock-in construct. The inserted Src1 sequences were amplified with linker primers D32 (ttcaggtaccaactaccaaaatagaacc)/D33 (gatcGTCGACATTTTCAAAAGAATTATTATTGAG) for the C-terminal coding sequence and D34 (ataataCTGCAGataataataataaaattaaaac)/ D35 (gaggGGATCCaaataaaaataaaaataaacc) for the 3′ untranslated sequence. For the mars-Src1 overexpression construct pIS1192, the GFP-Src1 vector pPB130 ^48^ was used and GFP was replaced with mRFPmars ^49^. For the GFP-CHMP7 construct, full-length sequence of CHMP7 (*DDB0266400*) was cloned into the N-terminal GFP overexpression construct pIS77 using *Bam*HI/*Sal*I restriction sites ^50^. For the mRFP-CHMP7 construct, GFP was replaced in this vector with mRFPmars.

**Antibodies**

Primary antibodies used in this study were monoclonal mouse anti-β-tubulin WA3 (kind gift from Ursula Euteneuer, LMU München), monoclonal rat YL1/2 directed against α-tubulin ^51^, polyclonal rabbit anti-GFP ^52^, polyclonal rabbit anti-Sun1^50^, polyclonal rat anti-Src1 ^48^, monoclonal anti-filactin 3S-55-4 ^53^, polyclonal rabbit anti-BirA (this paper). Secondary antibodies used for immunofluorescence were Alexa Fluor conjugates from Life Technologies (Darmstadt, Germany), and enzyme conjugates for Western blotting from Sigma (Deisenhofen, Germany).
